# Case Report: Integrated cardiovascular and respiratory training as a novel therapeutic approach in a case of painful left bundle branch block

**DOI:** 10.3389/fphys.2026.1793253

**Published:** 2026-05-01

**Authors:** Oscar Crisafulli, Venere Quintiero, Caio V. Spaggiari, Anna Odone, Giuseppe D’Antona

**Affiliations:** 1 Centtro di Ricerca Interdipartimentale Attività Motorie e Sportive (CRIAMS)-Sport Medicine Centre Voghera, University of Pavia, Voghera, Italy; 2 Cardiac Pacing Unit of the Heart Institute (InCor), Hospital das Clínicas, Faculty of Medicine, University of São Paulo (HCFMUSP), São Paulo, Brazil; 3 Department of Public Health, Experimental and Forensic Medicine, University of Pavia, Pavia, Italy; 4 Medical Direction, Fondazione Istituto di Ricovero e Cura a Carattere Scientifico (IRCCS) Policlinico San Matteo, Pavia, Italy

**Keywords:** aerobic training, case report, diaphragmatic breathing, heart rate modulation, individualized exercise prescription, painful left bundle branch block

## Abstract

**Background:**

Left bundle branch block (LBBB) is a heart rate (HR)–dependent cardiac conduction disorder that may occur in the absence of structural heart disease and can be associated with painful episodes. Evidence supporting exercise-based interventions in this setting is limited. We report the case of a 41-year-old woman with paroxysmal, painful LBBB, with onset occurring marginally above resting HR.

**Methods:**

After a diagnostic evaluation demonstrating preserved biventricular function, absence of myocardial ischemia, and normal hemodynamic responses, a 10-month supervised training program was implemented in three sequential phases: (1) diaphragmatic breathing exercise; (2) aerobic exercise performed below the individual LBBB HR threshold; (3) interval and steady-state aerobic exercise prescribed according to individually determined ventilatory thresholds (VT1 and VT2). Resting HR, HR at LBBB onset, maximal oxygen consumption (V˙O_2_max), ventilatory thresholds, and their associated HRs were longitudinally assessed.

**Results:**

After training, resting HR decreased by 15 bpm, while HR at LBBB onset increased by 50 bpm, markedly expanding the safe exertional window. V˙O_2_max improved progressively, together with upward shifts in VT1 and VT2, and reductions in corresponding HRs. Importantly, the patient reported resolution of LBBB-related pain during daily activities and even when LBBB was occasionally elicited at higher exercise intensities.

**Conclusions:**

This case suggests that a tailored respiratory and aerobic training program may safely improve cardiovascular efficiency, functional capacity, and symptom control in a patient with painful, HR-dependent LBBB. Individualized exercise training may represent a non-invasive adjunct or alternative to pharmacological or pacing strategies in selected patients.

## 1 Introduction

Left bundle branch block (LBBB) is a cardiac conduction abnormality characterized by delayed depolarization of the left ventricle, often associated with underlying structural heart disease, ischemic conditions, or cardiomyopathies (Tan et al., 2020). However, LBBB can also occur in individuals without apparent cardiac pathology, sometimes presenting with atypical symptoms, including chest pain (Shvilkin et al., 2016; Liszewski and Oskarsson, 2023). While LBBB is generally considered an incidental finding in asymptomatic patients, cases of painful LBBB, where patients experience chest discomfort in the absence of ischemic heart disease, have been reported (Seetharam et al., 2020; Liszewski and Oskarsson, 2023). The mechanisms underlying this phenomenon remain unclear. However, retrosternal pain is thought to arise from LBBB-induced left ventricle (LV) contraction dyssynchrony, possibly combined with endothelial dysfunction, small vessel spasm, and imbalance between regional myocardial workload and microvascular perfusion (Pestrea et al., 2025). Specifically, early work by Virtanen et al. (1982) linked uncoordinated heart motion to chest pain. Coherently, Higgins et al. (2006) demonstrated a link between perfusion defects and dyssynchronous LV contraction, reporting that delayed contraction of the LV lateral wall increases diastolic filling pressure, impairs subendocardial microvascular perfusion, and leads to post-systolic compression of septal perforators, ultimately compromising diastolic coronary flow during LBBB. Cardiac magnetic resonance imaging (MRI) studies support this mechanism, showing that delayed LV free wall contraction raises septal pressure and reduces coronary perfusion at end-systole (van der Land et al., 2007). Inoue et al. (2023) also suggested that microvascular dysfunction may contribute to painful LBBB, either through mechanical compression from dyssynchrony, chronic microvascular stress causing structural stenosis, or coincidental coexistence of LBBB and microvascular impairment. Additionally, direct viral damage to the myocardium or immune-mediated inflammation caused by COVID-19 have been proposed as possible causes (Kitsou et al., 2022). The management of painful LBBB is primarily pharmacological, with beta-blockers being the most used therapy to reduce symptoms by lowering heart rate (HR) and minimizing conduction abnormalities (Shlipak et al., 2000). However, in refractory cases, where pharmacological treatment fails to control symptoms, cardiac pacing, particularly His bundle pacing, is considered a viable option to restore a more physiological ventricular activation pattern (Ponnusamy et al., 2020). Beyond these conventional approaches, very few studies have evaluated the effect of exercise as a therapeutic strategy in patients with painful LBBB. To the authors’ knowledge, only one previous case report (Heinsimer et al., 1986) analyzed the effects of exercise training in this condition. In that study, a marked increase in the LBBB onset threshold was reported after the exercise intervention, which was associated with a progressive reduction of symptoms, and ultimately allowed discontinuation of beta-blocker therapy. However, in that case, LBBB onset occurred between 130 and 140 beats per minute (bpm), consistent with literature data showing that the threshold typically ranges from 120 to 140 bpm (Stein et al., 2011). Since the patient’s baseline HR was around 90 bpm, there was a sufficient margin to allow exercise without discomfort. When the onset threshold occurs close to resting HR, such an approach becomes impractical, as the patient would inevitably experience discomfort and pain during exercise. This scenario necessitates the exploration of complementary and/or alternative training strategies to enable sustainable physical exercise, with the ultimate goal of increasing the LBBB onset threshold and expanding the range of activities that can be performed without symptoms. Accordingly, this study explores the feasibility and potential effects of a personalized exercise approach in the presence of a low HR–dependent LBBB, a context that has not been addressed in previous literature and in which conventional exercise training is typically not applicable.

## 2 Case presentation

We report the case of a 41-year-old woman with paroxysmal, painful LBBB characterized by a low onset threshold, in the absence of significant past medical history or structural heart disease, who underwent a tailored, supervised training program aimed at optimizing cardiovascular efficiency, reducing symptoms, and enhancing exertion tolerance. In April 2023, the patient contracted severe acute respiratory syndrome coronavirus 2 (SARS-CoV-2), following which she began experiencing chest pain and was referred cardiology care. The patient subsequently underwent comprehensive clinical examinations, as detailed below.

### 2.1 Holter electrocardiogram 24h

The procedure was performed in August 2023. A Philips DigiTrak XT Holter monitor was used. The patient exhibited a mean resting HR of 70 bpm with sinus rhythm and intermittent LBBB, typically occurring at 75 bpm, along with isolated premature ventricular beats (PVBs) and isolated premature supraventricular beats (PSVBs). No alterations of the ST segment were observed, and no pauses were detected. A pronounced tachycardia occurred during physical exertion, as expected. These mean resting and trigger HR values were considered baseline measurements and are hereafter referred to as T0.

### 2.2 Myocardial perfusion tomoscintigraphy (gated single-photon emission computed tomography, G-SPECT)

The examination was performed in August 2023 over two days using the G-SPECT technique to assess myocardial perfusion and left ventricular kinetics at rest and during exercise stress. Of note, during the exercise stress, a HR of 180 bpm was achieved and the LBBB was reproduced. Images were reconstructed using an iterative algorithm, with slices oriented along the three main axes of the left ventricle for qualitative perfusion analysis. Seventeen-segment polar maps were generated using the Cedars-Sinai algorithm for semiquantitative analysis of perfusion and segmental kinetics. Qualitative evaluation revealed a mild, circumscribed, fixed perfusion defect in the mid-distal anteroseptal region, likely attributable to an attenuation artifact. Perfusion of the remaining left ventricular walls was homogeneous both at rest and under exercise stress. Semiquantitative analysis of the gated study demonstrated a left ventricular ejection fraction (LVEF) >70% at both rest and stress, with normal wall motion. Overall, the test was negative for exercise-induced myocardial ischemia, confirming normal left ventricular function.

### 2.3 Coronary computed tomography angiography

The procedure was performed in September 2023. Right coronary dominance was observed. The coronary tree was free from significant stenoses, and no anomalies in the origin of the coronary arteries were identified. The mid-anterior interventricular artery exhibited a superficial intramyocardial course (intramyocardial segment length: 2.5 cm; maximum depth <1 mm).

### 2.4 Stress cardiac magnetic resonance imaging

In September 2023, the procedure included acquisition of electrocardiogram (ECG)-gated bright blood cine sequences and perfusion imaging under pharmacological stress with Regadenoson (400 µg, single intravenous bolus). Inversion recovery (IR) sequences were used for delayed enhancement evaluation following intravenous administration of a paramagnetic contrast agent (Prohance, 0.2 mmol/kg). Both left and right ventricles exhibited preserved volumes and normal systolic function, with no wall motion abnormalities. Wall thickness and myocardial mass were within normal limits. Cardiac valves, atrial chambers, pulmonary artery, and thoracic aorta were normal in size and morphology. No pericardial effusion was detected. No myocardial perfusion defects or delayed enhancement were observed.

### 2.5 Ecocardiodoppler

The procedure was performed in November 2023, with the patient in sinus rhythm (SR) showing bundle branch block (BBB) morphology and septal dyssynchrony. The left ventricle (LV) showed normal dimensions and wall thickness, with an interventricular septum (IVS) thickness of 7 mm, diastolic diameter (DD) of 44 mm, posterior wall (PW) thickness of 7 mm, and indexed end-diastolic volume (EDVi) of 51 mL/m². First-degree diastolic dysfunction was present, with estimated filling pressures within normal limits. Atrial chambers were of normal size. The mitral valve demonstrated mild leaflet fibrosis with minimal regurgitation, while the tricuspid valve was normally functioning. The proximal ascending aortic root exhibited normal dimensions.

### 2.6 Right heart catheterization

In April 2024, the patient underwent right heart catheterization (RHC) at rest and during incremental exercise to exhaustion to evaluate circulatory and ventilatory responses to physical exertion. At baseline, pulmonary circulation and filling pressures were normal bilaterally. Cardiac output was within the normal-high range. No oxygen saturation differences suggestive of intracardiac shunt were detected. At peak exercise (lactate 6.8 mmol/L, respiratory quotient [RQ] 1.14), there were no signs of cardiorespiratory maladaptation. Filling pressures adapted normally bilaterally, with pulmonary arterial wedge pressure/cardiac output (PAWP/CO) of 1.3 mmHg/L/min and mean pulmonary arterial pressure/cardiac output (mPAP/CO) of 1.7 mmHg/L/min.

### 2.7 Cardiopulmonary exercise test

The procedure was performed in May 2024. CPET was performed on a cycloergometer (E 100, Cosmed, Italy), with the patient wearing a two-way breathing mask covering the nose and mouth (V2 Mask TM, Hans Rudolph Inc., United States), connected to a gas analyzer (Quark PFT, Cosmed, Italy). CPET was performed under ECG monitoring, with an incremental technique (12 W every 30 s, with a previous warm up of 3.5 min at 25 W), and V˙O2 and V˙CO2 output were measured using the breath-by-breath method. The test was considered maximal when it met three criteria: RER > 1.1, ratio of perceived exertion (RPE) ≥ 8 and V˙O2 slope at plateau for at least 30 s (Crisafulli et al., 2025). The test was performed until exhaustion, with the achievement of a respiratory exchange ratio (RER) of 1.22 and a RPE of 9. The maximal power reached was 145 watts. Normalized maximal oxygen consumption (V˙O_2_max) was 27 mL·kg^-^¹·min^-^¹ (normal range for age and gender over 26 mL·kg^-^¹·min^-^¹ according to Wasserman extended (Hansen et al., 1984). The HR peak was 174 bpm, 97% of the predicted value. First ventilatory threshold (VT1) (or gas exchange threshold, GET) was 15.44 mL·kg^-^¹·min^-^¹ (reached at 133 bpm) while the second ventilatory threshold (VT2) (or respiratory compensation point, RCP), was 21.97 mL·kg^-^¹·min^-^¹ (reached at 147 bpm). Of note, GET and RCP were assessed using Cosmed Omnia software. Specifically, GET was determined using the V-slope method by plotting V˙O_2_ vs V˙CO_2_, while RCP was identified by locating the increase in minute ventilation (V˙E)/V˙CO_2_ slope and the peak of end-tidal carbon dioxide partial pressure (PETCO_2_), and were independently verified by an experienced CPET interpreter (GD). These values were considered baseline measurements and are hereafter referred to as T0.

### 2.8 Clinical framework summary

Overall, the examinations revealed a HR-dependent LBBB, with no structural heart disease identified on resting transthoracic echocardiography (TTE) and no structural abnormalities or regional hypoperfusion detected on stress cardiac MRI. Exercise myocardial G-SPECT showed no evidence of ischemia. Holter ECG monitoring was negative for paroxysmal blocks or sinus dysfunction and demonstrated a normal circadian HR variation. RHC indicated normal hemodynamic adaptation to exercise. Of note, LBBB onset HR threshold was only marginally above the mean resting HR, with the conduction abnormality occurring even after minimal postural changes, substantially complicating management. Due to the persistence and severity of symptoms despite beta-blocker therapy, the patient was placed on a waiting list for His bundle pacing as a potential therapeutic option to restore physiological ventricular activation.

In October 2024, the patient came to our Sports Medicine Centre seeking alternative approaches to pacing therapy. She had a body weight of 67 kg and a height of 1.66 m, resulting in a body mass index (kg/m²) of 24.36. She reported being a non-smoker and to regularly assume beta blocker therapy (bisoprolol 1.25 mg x 2/day). The patient reported that this dose of bisoprolol was the maximum she could tolerate, as higher doses had previously caused hypotension. Previous pharmacological approaches also included ivabradine (5 mg/day) and nadolol (80 mg/day). However, these strategies proved unsustainable and were discontinued shortly thereafter. Specifically, nadolol was associated with a higher resting HR compared with bisoprolol, while ivabradine caused significant headaches. Following these attempts, pharmacological therapy was ultimately maintained as described above, and no further interventions were pursued.

Based on the findings obtained from the diagnostic assessments, she underwent a 10-month supervised training program, which was carefully tailored to her LBBB onset threshold and VTs determined through CPET. The objective was to increase the LBBB onset threshold and to achieve a progressive reduction in symptoms (Heinsimer et al., 1986). Of note, all along the training period the patient maintained the usual beta blocker therapy.

## 3 Training protocol and instrumental evaluations

### 3.1 Respiratory training

To implement a sustainable training regimen, it was essential to identify a mode of exercise that did not provoke pain or discomfort in the patient. The low HR at LBBB onset (75 bpm, compared with a mean resting HR of 70 bpm) posed a challenge, as even minimal exertion could trigger painful symptoms. Therefore, at this stage, we implemented a respiratory training protocol focused on diaphragmatic breathing with an asymmetry between the inspiratory and expiratory phases, technique that has previously been shown to reduce heart and respiratory rates, increase breath depth, optimize ventilatory efficiency, and minimize potential respiratory contributions to cardiac stress (Mason et al., 2013). Specifically, while lying in a supine position, the participant performed a slow breathing protocol at a rate of six breaths per minute. At the beginning of each session (for approximately 5 minutes), she performed 5-second inspiration and 5-second expiration cycles to become accustomed to the pacing and then progressed to 3-second inspiration and 7-second expiration cycles. No specific equipment was used during the training. The aim of this phase was to lower mean resting HR and widen the gap to the LBBB onset, thereby creating a range within which the patient could exercise comfortably. *S*uch modality was performed for two months, 3 times per week for 60 minutes per session. This exercise volume was considered sufficient to induce changes in HR, in accordance with previous literature (Laborde et al., 2022). In December 2024, the patient underwent a resting 12-lead ECG (Quark T12x, Cosmed, Italy) to evaluate mean resting HR (T1). Specifically, it was derived from a 10-s lead II recording obtained under standardized resting conditions. The patient was in the supine position after at least 5 minutes of quiet rest before ECG acquisition. Mean HR was calculated from the average RR interval over the 10-s recording (Soliman et al., 2010). Due to the observed reduction in mean resting HR, and consequently the increased HR gap between rest and LBBB onset, training was progressed to a cycling protocol, as detailed below.

### 3.2 Combination of respiratory and cycle ergometer training

After focusing exclusively on respiratory training, the patient engaged in a structured cycling ergometer program, with continuous ECG monitoring supervised by a medical doctor (GD), to check for cardiac events and ensure safety. The program consisted of three weekly sessions of one hour each, comprising sub-threshold LBBB onset aerobic training performed on a cycle ergometer. During such exercises, the patient was instructed to maintain the respiratory technique learned during the early training phase. This was aimed to further optimize ventilatory efficiency and reduce sympathetic activation (Laborde et al., 2022), minimizing potential triggers of LBBB-related symptoms. This approach continued for four months (between December 2024 and March 2025), a duration considered sufficient to elicit adaptations to aerobic exercise (Matsuo et al., 2014; Crisafulli et al., 2024).

**Figure 1:**
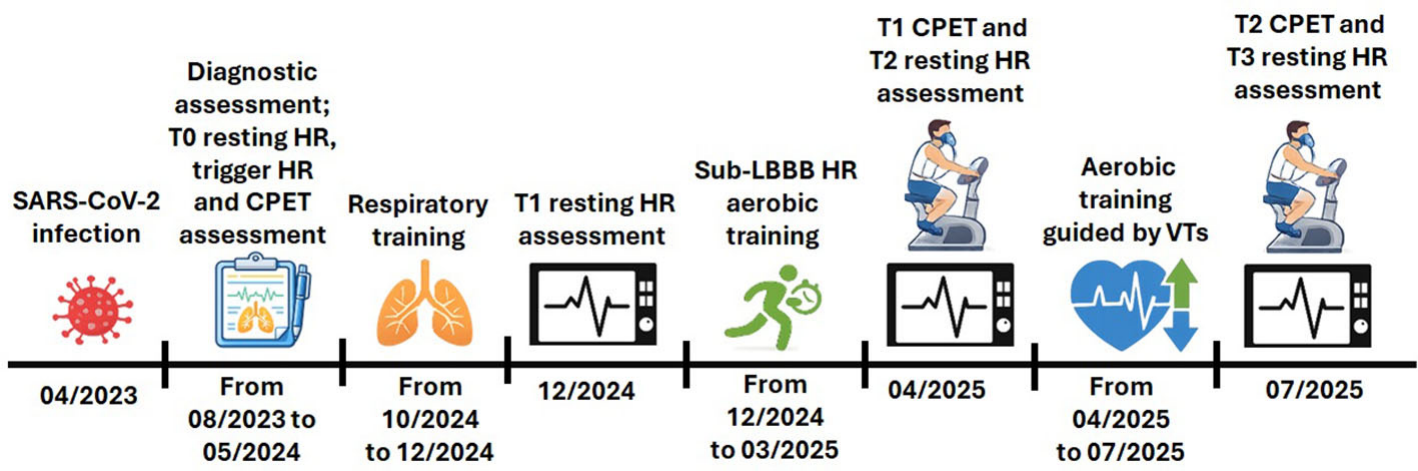
Timeline of case’s salient events. SARS-CoV-2, severe acute respiratory syndrome coronavirus 2; HR, heart rate; CPET, cardiopulmonary exercise test; LBBB, left bundle branch block; VTs, ventilatory thresholds.

Importantly, after this phase, the patient reported that the characteristic LBBB–related pain had resolved during many activities of daily living, including physically demanding tasks such as stair climbing and brisk walking. This prompted us to test a more intense exercise modality than previously used, while maintaining the option to return to the prior modality in case of exercise-induced pain. This exercise adaptation strategy was pursued based on evidence indicating that higher intensity aerobic exercise induces superior cardiovascular adaptations compared with lower intensity training (Helgerud et al., 2007; Ramírez-Vélez et al., 2020). These adaptations are known to translate into a reduction in the HR required for a given workload (Cornelissen et al., 2010), thereby potentially improving exertion tolerance. The objective was therefore to exercise at higher HRs in a controlled environment in order to efficiently reduce the HRs required for daily tasks and to lower the risk of LBBB occurrence during everyday activities. In line with evidence supporting a VT–based for prescribing exercise intensity in cardiac patients (Anselmi et al., 2021), an additional CPET was performed in April 2025 (T1) to determine VTs and inform the subsequent training phase. Of note, the test was performed following the same procedures as in the T0 assessment and ECG monitoring allowed also assessment of potential changes in the HR at LBBB onset. Specifically, VT1-based exercise was performed as steady-state training at an intensity approaching the threshold for 30 minutes. VT2-based exercise was delivered as interval training, consisting of 1 minute above the threshold (approximately 85–90% of maximal intensity) followed by 2 minutes below the threshold. This sequence was repeated 10 times per session, resulting in a total of 10 minutes above and 20 minutes below the threshold. Frequency and duration of the sessions remained unchanged. These exercise modalities were selected to progressively enhance cardiovascular efficiency (Ghosh, 2004). Such approach continued for four months. After this phase, in July 2025, the patient underwent a follow-up CPET to assess post-training cardiopulmonary changes (T2) and potential changes in the HR at LBBB onset. Again, the test was performed following the same procedures of T0. In both April and July 2025, alongside the CPET, ECG assessments were performed to evaluate basal HR (T2 and T3, respectively), following the same procedure described for the T1 assessment. A graphical representation of the timeline of the case’s salient events, including assessments and training phases, is shown in **Figure 1**.

## 4 Results and discussion

**Figure 2:**
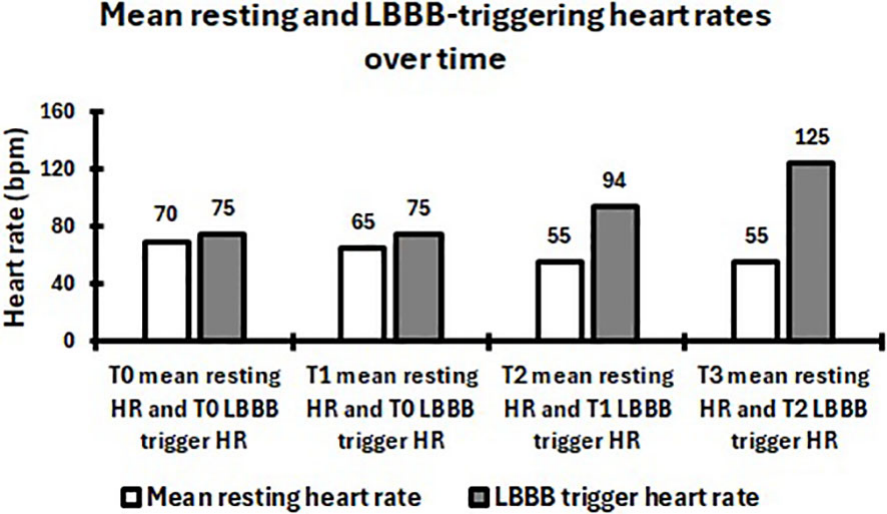
Mean resting and LBBB onset heart rates. White bars indicate resting heart rate, whereas gray bars indicate LBBB onset heart rate.

In this case report, we describe a patient with painful, HR–dependent LBBB occurring in the absence of structural heart disease, inducible ischemia, or abnormal cardiovascular adaptation to exercise, as confirmed by an extensive diagnostic evaluation. The condition was characterized by an exceptionally low HR threshold for LBBB onset, only marginally above resting values, and was successfully managed with a tailored, supervised training program. Within this framework, the intervention was deliberately structured in three sequential phases: an initial phase focused on respiratory training to reduce mean resting HR and expand the physiological buffer below the LBBB onset threshold, followed by a second phase incorporating LBBB onset HR oriented aerobic training, and a third phase of intensified aerobic training guided by VTs. The initial respiratory training phase has presumably played a pivotal role in enabling subsequent exercise. Controlled diaphragmatic breathing with prolonged expiration has been shown to reduce resting HR, enhance parasympathetic tone, improve ventilatory efficiency, and attenuate sympathetic drive (Mason et al., 2013; Hamasaki, 2020). In this patient, such an approach appears to have led to a clinically meaningful reduction in mean resting HR from 70 to 65 bpm (**Figure 2**), consequently widening the gap between rest and LBBB HR onset, ultimately creating a physiological window in which aerobic exercise could be safely introduced.

Following the phase of sub-LBBB-threshold aerobic cycling and continued respiratory control, several cardiorespiratory improvements were observed. Specifically, the T1 CPET revealed that, compared to T0, V˙O_2_max, peak power output, VT1, and VT2 increased by 1.10 mL·kg^-^¹·min^-^¹, 8 W, 2.29 mL·kg^-^¹·min^-^¹, and 1.11 mL·kg^-^¹·min^-^¹, respectively. Moreover, HR recorded during exercise phases were lower, with a reduction of 5 bpm at peak, 2 bpm at VT1, and 3 bpm at VT2 (**Table 1**). Of note, as at T0, the test met the predefined criteria for maximal effort, with a RER of 1.19 and an RPE of 9. Overall, although a direct causal relationship cannot be established, these results appear to be consistent with the literature regarding the effects of low−intensity aerobic training (Nuuttila et al., 2026). Notably, mean resting HR decreased from 65 to 55 bpm, while the trigger HR for the LBBB increased from 75 to 94 bpm (**Figure 2**), thereby further enlarging the physiological subthreshold buffer.

**Table 1:** CPET results.

CPET parameters	T0	T1	T2	T0-T1 absolute changes	T1-T2 absolute changes	T0-T2 absolute changes
V˙O_2_max (mL·kg^-^¹·min^-^¹)	27	28,1	31	1,1	2,9	4
HR peak (bpm)	174	169	158	-5	-11	-16
Maximal Power (W)	145	153	169	8	16	24
VT1 (mL·kg^-^¹·min^-^¹)	15,44	17,73	23,1	2,29	5,37	7,66
HR at VT1 (bpm)	133	131	128	-2	-3	-5
VT2 (mL·kg^-^¹·min^-^¹)	21,97	23,08	26,1	1,11	3,02	4,13
HR at VT2 (bpm)	147	144	138	-3	-6	-9

V˙O_2_max, maximal oxygen consumption; HR, heart rate; bpm, beats per minute; VT1, first ventilatory threshold; VT2, second ventilatory threshold; CPET, cardiopulmonary exercise test.

It is worth noting that, as previously mentioned, after this phase the patient reported progressive symptom improvement and eventual resolution of pain during activities of daily living that had previously elicited symptoms. This observation led us to hypothesize that a training-induced upward shift of the LBBB onset threshold (Heinsimer et al., 1986), together with increased cardiac efficiency and a consequently lower HR response to exertion (Zavorsky, 2000), may have resulted in daily tasks being performed below threshold and therefore not triggering the block. However, during the T1 CPET, despite the LBBB being elicited by the high HR inherent to the maximal nature of the test, the patient reported no pain. This prompted further hypotheses on the theoretical mechanisms behind the patient-reported resolution of pain during daily activities, suggesting that, in addition (or alternatively) to the increased trigger threshold and reduced exercise HR, the patient may have tolerated block-inducing HRs without experiencing pain. The possible physiological mechanisms underlying this phenomenon remain unclear, as the factors responsible for chest pain in these patients are also not fully understood (Silva et al., 2025). For instance, Virtanen et al. (1982) proposed as a possible explanation that altered cardiac motion may be perceived by the patient as chest discomfort. Subsequently, Heinsimer et al. (1986) suggested that the symptoms could be attributable to microvascular ischemia. On a theoretical basis, the present case could align with this latter hypothesis, as aerobic training is well known to promote microvascular remodeling, thereby enabling an increased oxygen supply to the myocardium (Koller et al., 2022); adaptations which could counteract microvascular ischemia and ultimately lead to a reduction or complete resolution of pain. While our results do not allow us to draw definitive conclusions, they highlight a physiological issue that deserves further investigation in future studies.

**Figure 3:**
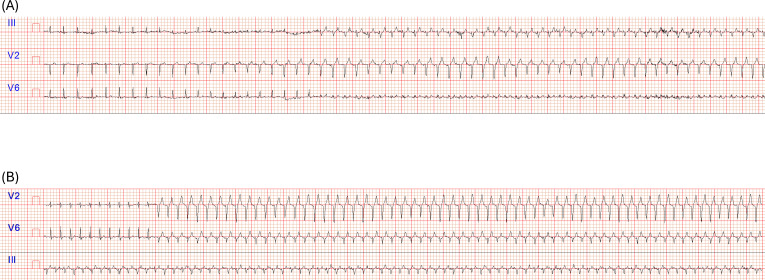
Representative ECG tracings illustrating the heart rate–dependent onset of LBBB at T1 **(A)**, and T2 **(B)**.

However, the absence of discomfort reported by the patient prompted us to test a higher-intensity, VT-based exercise modality in order to further enhance cardiovascular capacity (Martini et al., 2022) and, potentially, to increase exertion tolerance. Coherently with our expectations, T2 results suggest that VTs–based training may have further enhanced cardiovascular efficiency (Guio de Prada et al., 2019; Anselmi et al., 2021) while maintaining symptom control. In fact, respect to T1, a further increase in V˙O_2_max was observed (+2.9 mL·kg^-^¹·min^-^¹). Maximal power output increased of 16 W, VT1 increased of 5.37 mL·kg^-^¹·min^-^¹ and VT2 of 3.02 mL·kg^-^¹·min^-^¹. In addition, peak HR decreased by 11 bpm, as did HR at both VT1 and VT2 (-3 and -6 bpm, respectively), while the LBBB threshold further increased, rising from 94 to 125 bpm. In this case as well, the test met the predefined criteria for maximal effort, with a RER of 1.17 and an RPE of 9. The ECG tracings strips showing the different LBBB onset HRs after aerobic training phases are presented in **Figure 3**. Notably, these trends were already appreciable in the comparison between T0 and T1; however, comparisons between evaluations suggest that the largest changes occurred after VT-based training. All CPET data, including the deltas between assessments, are reported in **Table 1**. Of note, the patient occasionally developed LBBB during training sessions, particularly during VT2-based phases. These episodes were not systematically quantified in terms of frequency or duration; however, as also observed during the T1 CPET, she never reported pain. Although exercise prescription based on VTs is now well established in sports medicine and cardiac rehabilitation (Anselmi et al., 2021), its application in patients with painful LBBB has not previously been described and may represent a novel and clinically relevant approach.

Besides, resting ECG assessments revealed that basal HR did not change following the VT-based exercise modality (**Figure 2**), although a reduction would have been physiologically plausible (Anselmi et al., 2021). The absence of a further decrease may reflect a physiological floor effect (Reimers et al., 2018), as resting values would have already been substantially reduced after both the respiratory and sub-LBBB-threshold aerobic training interventions.

The temporal association between symptom onset and prior SARS-CoV-2 infection is also noteworthy. Although no imaging evidence of myocarditis or myocardial fibrosis was detected, emerging data suggest that COVID-19 may be associated with new-onset LBBB or other conduction disturbances, although the underlying mechanisms remain to be clarified (Shoura et al., 2023). While causality cannot be established, viral- or immune-mediated effects on the conduction system may have contributed to the onset of the LBBB found in the patient (Kitsou et al., 2022).

Although the study design does not allow for a causal relationship to be established, from an exercise physiology perspective, this case would point out the possible role of autonomic modulation and cardiovascular efficiency in shaping HR–dependent conduction abnormalities. Clinically, it suggests that individualized, supervised training programs incorporating respiratory and VT based aerobic training may serve as a useful adjunct to pharmacological therapy in selected patients with painful LBBB. Such an approach may have the potential to improve functional capacity, reduce symptom burden, and, in some cases, delay or obviate the need for invasive pacing strategies. Further cohort’s studies are warranted to confirm or refute the hints of this case report, with particular reference on the physiological mechanisms underlying pain resolution and the increase in LBBB onset threshold.

## 5 Limitations and future directions

Some limitations warrant consideration. As a single-case report, the findings cannot be generalized, and spontaneous variability in symptoms cannot be excluded. While the observed improvement likely reflects the effects of training, we cannot rule out the possibility that the pain resolution occurred as part of the natural course of the condition in this individual. In addition, baseline rest and LBBB onset HRs were derived from 24-hour Holter monitoring under non-standardized conditions, which may limit the reproducibility of the threshold and its comparability with post-intervention assessments. Although never accompanied by pain or requiring specific management strategies, the LBBB episodes observed during VT-based training were not systematically recorded. Therefore, information regarding their frequency, HRs at onset, duration, and progression is not available, which precludes drawing definitive conclusions regarding safety and symptom control. Pain evaluation was based on patient self-report during the intervention period, and therefore the interpretation of the clinical effect should be considered descriptive rather than quantitative, possibly limiting the interpretability of results. Moreover, although symptomatic improvement was evident, objective reassessment of the clinical manifestations of LBBB after deconditioning has not been investigated. Future studies on a wide cohort, incorporating a longer follow-up both considering long lasting exercise training, or its interruption are needed to better define the real significance of the observed clinical outcomes and to provide clinicians and patients with the correct approach to manage the disease.

## 6 Conclusion

This case highlights the diagnostic challenges of painful LBBB and suggests, for the first time, that a respiratory-aerobic based exercise program can serve as a safe and effective complementary strategy. It underscores the importance of differentiating painful LBBB from other causes of chest pain and, suggests that targeted training may offer a noninvasive alternative, or adjunct, to His bundle pacing therapies in selected patients, thereby opening new avenues for future research in this field.

## Data Availability

The original contributions presented in the study are included in the article/supplementary material. Further inquiries can be directed to the corresponding author.
